# Supplementary material for: Trends, geographic distribution, and disease burden of bipolar disorder in Ecuador (2011–2021): An analysis of hospital discharge data
Source: PLoS One. 2025 May 23;20(5):e0320321. doi: 10.1371/journal.pone.0320321 (PMC12101731; doi:10.1371/journal.pone.0320321)
Supplement: S1Table — (DOCX) [file pone.0320321.s001.docx]

S1 Table. Parameters for DALY calculation

| Parameter | Probability distribution | Value range | | Source |
| --- | --- | --- | --- | --- |
| Population | Fixed by age and sex | 15.0 in 2010 – 17.8  million in 2021 inhabitants | | INEC (2023b) |
| Prevalence of Schizophrenia | Fixed | 0 to 8.6 per 100,000 people | | Calculated from data consolidated by INEC  (2023a) |
| Disability Weight: Weighted average of mild, moderate and severe cases (9%, 61%  and 30%, respectively). | Fixed | Weighted average DW  = 0.496 | | Calculated from data taken from Kim et al (2021). |
| Assumed average duration of disability in years (males and  females). | Fixed | 1 year | | Assumed due to unknown duration of illness. |
| Treatment proportion of male and female | Fixed | 1 [range 0 – 1]. | | Due to data being used relating entirely to  treated cases. |
| INCIDENCE BY AGE GROUP (per 100,000 in  total population | | Male | Female |  |
| Age group 0 – 4 years | Fixed | 0 .00 | 0.01 | Calculated from data consolidated by INEC  (2023ba and 2023b) |
| Age group 5 – 14 years | Fixed | 0.11 | 0.23 | Calculated from data consolidated by INEC  (2023a and 2023b) |
| Age group 15 – 44 years | Fixed | 3.08 | 5.80 | Calculated from data consolidated by INEC  (2023a and 2023b) |
| Age group 45 – 59 years | Fixed | 4.80 | 8.60 | Calculated from data consolidated by INEC  (2023a and 2023b) |
| Age group 60+ years | Fixed | 3.28 | 5.44 | Calculated from data  consolidated by INEC (2023a and 2023b) |
