# Supplementary material for: Trends, geographic distribution, and disease burden of bipolar disorder in Ecuador (2011–2021): An analysis of hospital discharge data
Source: PLoS One. 2025 May 23;20(5):e0320321. doi: 10.1371/journal.pone.0320321 (PMC12101731; doi:10.1371/journal.pone.0320321)
Supplement: S2 Table — (DOCX) [file pone.0320321.s002.docx]

S2 Table. Female cases of bipolar disorder and incidence by year

| **Year** | **Number of cases** | **Persons-time at risk** | **Incidence rate in 100,000 person-years** | **Poisson confidence intervals at 95%** |
| --- | --- | --- | --- | --- |
| 2010 | 317 | 7568353 | 4.19 | [3.74;4.68] |
| 2011 | 310 | 7698755 | 4.03 | [3.59;4.5] |
| 2012 | 308 | 7829061 | 3.93 | [3.51;4.4] |
| 2013 | 277 | 7958814 | 3.48 | [3.08;3.92] |
| 2014 | 348 | 8087914 | 4.3 | [3.86;4.78] |
| 2015 | 391 | 8216234 | 4.76 | [4.3;5.25] |
| 2016 | 362 | 8343760 | 4.34 | [3.9;4.81] |
| 2017 | 439 | 8470420 | 5.18 | [4.71;5.69] |
| 2018 | 457 | 8596147 | 5.32 | [4.84;5.83] |
| 2019 | 500 | 8720919 | 5.73 | [5.24;6.26] |
| 2020 | 328 | 8844706 | 3.71 | [3.32;4.13] |
| 2021 | 361 | 8978739 | 4.02 | [3.62;4.46] |
| Total | 4398 |  |  |  |
| Yearly Mean Incidence |  |  | 4.43 | [4.3;4.56] |
