# Supplementary material for: Trends, geographic distribution, and disease burden of bipolar disorder in Ecuador (2011–2021): An analysis of hospital discharge data
Source: PLoS One. 2025 May 23;20(5):e0320321. doi: 10.1371/journal.pone.0320321 (PMC12101731; doi:10.1371/journal.pone.0320321)
Supplement: S3 Table — (DOCX) [file pone.0320321.s003.docx]

S3 Table. Cases and incidence of bipolar disorder in male Ecuadorians by year.

| **Year** | **Number of cases** | **Persons-time at risk** | **Incidence rate in 100,000 person-years** | **Poisson confidence intervals at 95%** |
| --- | --- | --- | --- | --- |
| 2010 | 196 | 7443875 | 2.63 | [2.28;3.03] |
| 2011 | 183 | 7567676 | 2.42 | [2.08;2.80] |
| 2012 | 187 | 7691912 | 2.43 | [2.10;2.81] |
| 2013 | 179 | 7815935 | 2.29 | [1.97;2.65] |
| 2014 | 212 | 7939552 | 2.67 | [2.32;3.05] |
| 2015 | 175 | 8062610 | 2.17 | [1.86;2.52] |
| 2016 | 208 | 8184970 | 2.54 | [2.21;2.91] |
| 2017 | 248 | 8306557 | 2.99 | [2.63;3.38] |
| 2018 | 251 | 8427261 | 2.98 | [2.62;3.37] |
| 2019 | 227 | 8547067 | 2.66 | [2.32;3.02] |
| 2020 | 162 | 8665937 | 1.87 | [1.59;2.18] |
| 2021 | 195 | 8870400 | 2.20 | [1.90;2.53] |
| Total | 2423 |  |  |  |
| Yearly Mean Incidence |  |  | 2.48 | [2.39;2.59] |
