# Supplementary material for: Trends, geographic distribution, and disease burden of bipolar disorder in Ecuador (2011–2021): An analysis of hospital discharge data
Source: PLoS One. 2025 May 23;20(5):e0320321. doi: 10.1371/journal.pone.0320321 (PMC12101731; doi:10.1371/journal.pone.0320321)
Supplement: S4 Table — (DOCX) [file pone.0320321.s004.docx]

S4 Table. Spatial clusters of BD in Ecuador

| **Cluster** | **Province(s)**  **Location** | **Radius in Km** | **Relative Risk** | **p-value** |
| --- | --- | --- | --- | --- |
| 1 | Pichincha | Single spot | 3.21 | < 0.0001 |
| 2 | Guayas | Single spot | 1.47 | < 0.0001 |
| 3 | Imbabura | Single spot | 1.84 | < 0.0001 |
| 4 | Azuay | 30.61 km | 1.42 | < 0.0001 |
| 5 | Chimborazo | Single spot | 1.55 | 0.00037 |
